# A Bioengineered Peptide that Localizes to and Illuminates Medulloblastoma: A New Tool with Potential for Fluorescence-Guided Surgical Resection

**DOI:** 10.7759/cureus.207

**Published:** 2014-09-17

**Authors:** Shelley E. Ackerman, Christy M. Wilson, Suzana A. Kahn, James R. Kintzing, Darren A. Jindal, Samuel H. Cheshier, Gerald A. Grant, Jennifer R. Cochran

**Affiliations:** 1 Bioengineering, Stanford University; 2 School of Medicine, Stanford University; 3 Institute for Stem Cell Biology and Regenerative Medicine, Stanford University School of Medicine, Stanford, CA 94305; 4 Department of Bioengineering, Stanford University; 5 Department of Bioengineering, Stanford University; 6 Department of Neurosurgery and Neurology, Stanford University School of Medicine & Lucile Packard Children’s Hospital, Department of Neurosurgery and Neurology, Stanford University School of Medicine & Lucile Packard Children’s Hospital at Stanford; 7 Department of Neurosurgery, Stanford University School of Medicine; 8 Department of Bioengineering and (by courtesy) Chemical Engineering, Stanford University

**Keywords:** knottin, cystine-knot peptide, tumor targeting, molecular imaging, integrin, fluorescence-guided surgical resection, medulloblastoma, engineered peptide, protein engineering, General Surgery, Neurosurgery, Oncology

## Abstract

Tumors of the central nervous system are challenging to treat due to the limited effectiveness and associated toxicities of chemotherapy and radiation therapy. For tumors that can be removed surgically, extent of malignant tissue resection has been shown to correlate with disease progression, recurrence, and survival. Thus, improved technologies for real-time brain tumor imaging are critically needed as tools for guided surgical resection. We previously engineered a novel peptide that binds with high affinity and unique specificity to α_V_β_3_, α_V_β_5_, and α_5_β_1_ integrins, which are present on tumor cells, and the vasculature of many cancers, including brain tumors. In the current study, we conjugated this engineered peptide to a near infrared fluorescent dye (Alexa Fluor 680), and used the resulting molecular probe for non-invasive whole body imaging of patient-derived medulloblastoma xenograft tumors implanted in the cerebellum of mice. The engineered peptide exhibited robust targeting and illumination of intracranial medulloblastoma following both intravenous and intraperitoneal injection routes. In contrast, a variant of the engineered peptide containing a scrambled integrin-binding sequence did not localize to brain tumors, demonstrating that tumor-targeting is driven by specific integrin interactions. *Ex vivo* imaging was used to confirm the presence of tumor and molecular probe localization to the cerebellar region. These results warrant further clinical development of the engineered peptide as a tool for image-guided resection of central nervous system tumors.

## Introduction

The quality of life and overall life expectancy of patients diagnosed with malignant brain tumors has not improved, despite significant advances made in the development of new cancer therapies [1–2]. Brain tumors, such as medulloblastoma, the most common malignant pediatric brain tumor, can be surgically resected with the extent of resection correlating with recurrence, metastasis, and overall survival rate [3–6]. Magnetic resonance imaging (MRI) is currently used by neurosurgeons for preoperative navigational planning; however, its usefulness is limited by brain movement that occurs during the procedure [7–8]. There is a critical need for real-time image-guided intraoperative approaches that would allow tumor boundaries to be effectively visualized and accurately demarcated during neurosurgery. Such tools have shown great promise in the clinic, to help guide surgeons in removing as much malignant tissue as safely possible while sparing the surrounding healthy tissue [7, 9].

**FIGURE 1:**
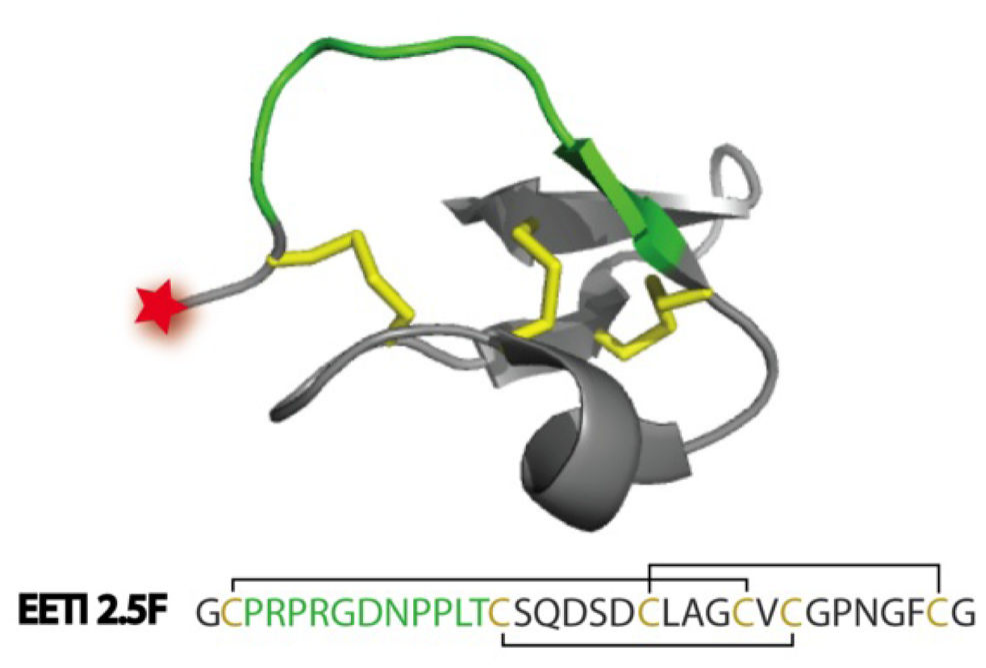
Knottin structure and sequence Three dimensional structure of wild-type EETI-II (PDB 2IT7). Red star indicates the location where an Alexa Fluor dye is conjugated to the peptide N-terminus. The primary sequence of EETI 2.5F is shown. Green: integrin-binding sequence, which has replaced the native EETI-II trypsin binding loop. Yellow: disulfide bonds.

To develop a fluorescent tumor-targeting agent as a real time imaging and navigational tool for improved surgical resection, we took a peptide that is found within the seeds of the squirting cucumber, *Ecballium elaterium,* that naturally functions as a trypsin enzyme inhibitor (known as EETI-II) [10], and used molecular engineering approaches to create an altered peptide that selectively binds to particular tumor-associated integrin receptors [11]. Integrins are cell adhesion receptors that have attracted great interest as tumor targets for cancer therapy and molecular imaging [12–14]. Specifically, α_V_β_3_, α_V_β_5_, and α_5_β_1_ integrins are expressed at high levels on tumor cells or tumor vasculature in a variety of cancers [15–16], and play important roles in angiogenesis, invasion, and metastasis [17–21]. EETI-II is part of a family of peptides consisting of three interlocking disulfide bonds that form a structural motif known as a cystine-knot (also called a knottin) (Figure 1) [22–23]. This disulfide-bonded framework confers high thermal, chemical, and proteolytic stability [24–25], which are ideal properties for diagnostic and therapeutic applications.

To engineer a tumor targeting agent based on EETI-II, we generated a library of millions of peptide variants in which the 6-amino acid trypsin binding loop of EETI-II was mutated to an 11-amino acid loop containing an Arg-Gly-Asp (RGD) integrin anchoring sequence [11]. This library of EETI-II peptides was displayed on the surface of yeast cells and screened using high-throughput techniques to identify variants that bound with high affinity to tumor-associated integrin receptors. The resulting lead candidate, termed EETI 2.5F, bound to α_V_β_3_, α_V_β_5_, and α_5_β_1_ integrins with tight affinities in the low nanomolar range [11]. Radiolabeled versions of EETI 2.5F were shown to have promise as PET imaging agents in a variety of mouse xenograft and genetic models due to their high tumor contrast and low imaging signals in non-diseased tissue, including the liver and kidneys [26–28]. More recently, we demonstrated that EETI 2.5F can specifically localize to intracranial murine medulloblastoma in genetic models of the disease, and can be used to illuminate brain tumor tissue when conjugated to a fluorescent probe [29]. Here, in the present study, we demonstrate that EETI 2.5F can selectively target and illuminate human medulloblastoma tissue in patient-derived xenograft models. This work provides the foundation necessary for potential clinical translation of EETI 2.5F in image-guided surgical resection of human brain tumors.

## Materials And Methods

### Medulloblastoma tissue collection and dissociation

A medulloblastoma tissue sample was obtained after appropriate patient consent at the Lucille Packard Children’s Hospital (Stanford, CA), in accordance with Institutional Review Board protocols (Protocol ID #: 12625; IRB #: 4593 (Panel: 5)). Pathology of the tumor was confirmed upon histopathologic analysis by institutional neuropathologists prior to further analysis.

The tumor sample was mechanically dissociated in a solution containing Hank’s Balanced Salt Solution (HBSS), nonessential amino acids, sodium pyruvate, sodium bicarbonate, HEPES, Glutamax-1, antibiotic-antimycotic, DNase, and collagenase IV. All media components were from Cellgro, except for DNase and collagenase IV, which were from Worthington. The suspension was washed two times with HBSS, filtered through a 70-μm strainer and resuspended in a 0.9 M sucrose solution in HBSS without Ca^2+^/Mg^2+^ (Cellgro) to remove debris and dead cells. The cells were treated with ACK/RBC lysis buffer (Gibco), then washed twice in phosphate buffered saline (PBS). Cells were plated in tumor stem media (TSM) consisting of Neurobasal-A medium (Invitrogen), B27-A (Invitrogen), human bFGF (20 ng/ml) (Shenandoah Biotech), human EGF (20 ng/ml) (Shenandoah Biotech), human recombinant LIF (20 ng/ml) (Millipore), and heparin (10 ng/ml).

### Peptide synthesis and fluorescent labeling

Linear knottin peptide sequences were constructed using solid-phase peptide synthesis on a CS Bio (Menlo Park, CA) instrument using 9-fluorenylmethyloxycarbonyl chemistry. Sequences of knottin peptides were EETI 2.5F: GCPRPRGDNPPLTCSQDSDCLAGCVCGPNGFCG or EETI RDG: GCVTGRGDSPASSCSQDSDCLAGCVCGPNGFCG. Peptides were folded as previously described [11, 27] by incubating in an oxidizing buffer with rocking at room temperature overnight to promote disulfide bond formation. Reversed-phase HPLC was used to separate unfolded or misfolded peptide from the folded product. Molecular masses of the folded knottin peptides were determined using matrix-assisted laser desorption/ionization time-of-flight (MALDI-TOF) mass spectrometry (Stanford Protein and Nucleic Acid Facility). For fluorescent-probe conjugation, knottin peptides were incubated with an amine-reactive succinimidyl ester derivative of Alexa Fluor 488 (AF488) or Alexa Fluor 680 (AF680) in 0.1 M sodium bicarbonate, pH 8.3, at a 1:5 peptide to dye molar ratio. The reaction proceeded at room temperature with stirring for two hours and then was incubated overnight at 4° C. Dye-conjugated knottins were purified using reversed-phase HPLC, and masses were confirmed by electrospray ionization (ESI) mass spectrometry (Stanford Mass Spectrometry Facility). All labeled peptides were dissolved in PBS, passed through a 0.22-μm filter, and concentrations were quantified by NanoDrop (Thermo Scientific) prior to animal experiments.

### Integrin expression

Integrin expression profiling was performed with 4×10^4^ MB-004 cells per reaction. Cells were pelleted and resuspended in 50 μL of a 1:100 dilution of anti-α_V_β_3_ (Abcam), 1:100 dilution of anti-α_V_β_5_ (Millipore), 1:25 dilution of anti-α_5_-FITC, or 1:25 dilution of anti-β_1_-AF488 (BioLegend) antibodies. Cells were incubated with primary antibodies for 40 min on ice and were then washed in PBS containing 0.1% bovine serum albumin (PBS/BSA). Cells treated with unlabeled antibodies were then incubated with goat anti-mouse IgG-FITC (Sigma) for 40 min and washed in PBS/BSA. Cells were analyzed on a Guava EasyCyte flow cytometer (EMD Millipore), and the resulting data was analyzed using FlowJo software (TreeStar).

### Tumor implantation

Animal procedures were carried out according to a protocol approved by the Stanford University Administrative Panels on Laboratory Animal Care (APLAC #28701 and APLAC #26548). NOD scid gamma (NSG) mice (four to eight weeks of age) were housed and bred in the Stanford University animal facility. For all imaging studies n=5; EETI 2.5F (n=3) and RDG negative control group (n=2). For tumor cell implantation, female NSG mice were anesthetized with 2.5% isoflurane by inhalation, with a flow rate of 1 L/min. 3×10^4^ MB-004 cells were stereotactically injected 2 mm posterior to the lambda and 2 mm deep with respect to the skull into the cerebellum of the mouse. Tumors were grown between one to two weeks prior to performing experiments. The presence of tumor was confirmed by visual inspection and bright field *ex vivo* imaging at the conclusion of the experiment.

### Molecular imaging

Prior to imaging, any fur on the head of the mouse was removed. Mice were anesthetized with isoflurane and injected with 1.5 nmol of either AF680-EETI 2.5F or the negative control probe AF680-EETI RDG in 100 μL of PBS via tail vein injection or intraperitoneal administration. Mice were imaged at zero, two, and 24 hrs post-administration using an IVIS Spectrum Series *In Vivo* Imaging System (Caliper Life Sciences). The AF680 near-infrared fluorophore was detected using an excitation wavelength of 630 nm and emission signal was monitored at 720 nm. Background autofluorescence was measured using an excitation wavelength of 535 nm and emission was detected at 720 nm. All optical imaging analysis was performed using Living Image software (Caliper Life Sciences). For *ex vivo* analysis, mice were euthanized two hrs post-administration, and brains were excised and imaged using the same excitation and emission settings as for *in vivo* imaging experiments. Whole brains were also cut along the midsagittal plane and imaged using identical settings to visualize binding specificity to the tumor within the brain. Representative images are shown in figures.

### Tissue sectioning and H&E staining

Formalin-fixed paraffin-embedded brain tissue was sectioned at 5 μm using a microtome and mounted on poly-1-lysine-coated glass slides. Hematoxylin and eosin (H&E) stained sections were imaged using a 2.5X objective on a Zeiss Axioplan 2 Epifluorescent microscope equipped with DIC optics at fixed exposure across tumor areas. Representative images are shown in figures.

## Results

### Patient-derived medulloblastoma cells express integrin receptors

**FIGURE 2:**
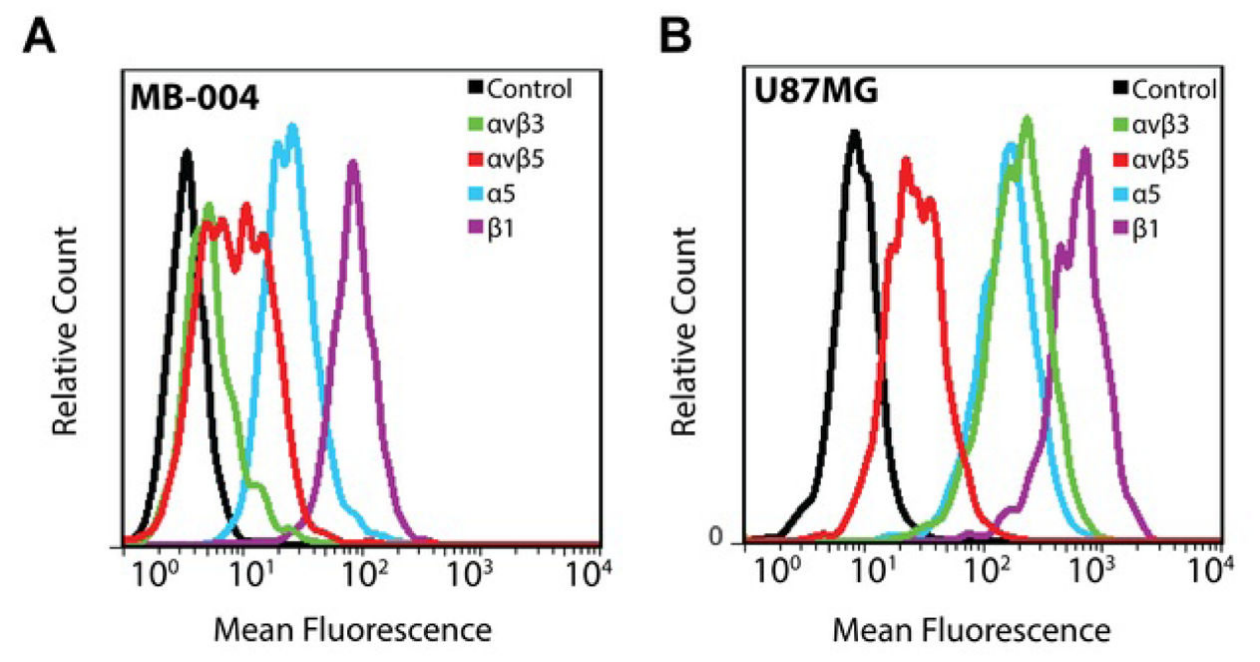
Integrin expression profiling of MB-004 and U87MG cells (A) Flow cytometry analysis of integrin expression on the surface of human medulloblastoma MB-004 cells. (B) As a positive control, integrin expression is shown in human glioblastoma U87MG cells.

Prior to assessing tumor targeting in an orthotopic murine model, we tested the integrin expression profile of MB-004, a patient-derived tumor cell line. MB-004 cells bound to an antibody specific for the α_V_β_5_ integrin heterodimer, as detected by flow cytometry using a fluorescein-conjugated secondary antibody, with marginal staining observed for an antibody specific for the α_V_β_3_ integrin heterodimer (Figure 2A). In addition, using fluorophore-conjugated primary antibodies, we showed that MB-004 cells expressed the α_5_ or β_1_ integrin subunit on their surface (Figure 2A). The α_5_ integrin subunit has only been found to partner with the β_1_ subunit, thus measurement of α_5_ indicates the presence of α_5_β_1_ integrin. Integrin expression profiling of the U87MG glioma cell line was performed as a control to validate the antibodies used in this experiment (Figure 2B), and was consistent with previously reported results [30]. This data shows that integrin receptors α_V_β_5_ and α_5_β_1_ are present on the surface of MB-004 human medulloblastoma cells, compared to minimal expression of α_V_β_3_ integrin.

### Chemical synthesis of dye-conjugated knottin EETI 2.5F and control peptide

The linear sequence corresponding to the EETI 2.5F peptide was produced by solid-phase peptide synthesis. The peptide was folded *in vitro* to form the disulfide-bonded structured knottin, which was purified by reversed-phase high-pressure liquid chromatography (RP-HPLC). The ability of folded knottin to bind to MB-004 cells was confirmed using flow cytometry to detect fluorescence of EETI 2.5F that was labeled with Alexa Fluor 488 (AF488) (data not shown). As a control, a knottin peptide containing a scrambled integrin-targeting sequence (AF488-EETI RDG) [11], did not exhibit binding to MB-004 cells (data not shown). For *in vivo* imaging experiments, a near-infrared fluorescent dye (Alexa Fluor 680, AF680) was conjugated to the N-terminal amine of EETI 2.5F or the control knottin EETI-RDG using succinimide ester chemistry, and the resulting labeled knottins were purified using RP-HPLC.

### Knottin EETI 2.5F targets intracranial medulloblastoma in patient-derived xenograft models

**FIGURE 3:**
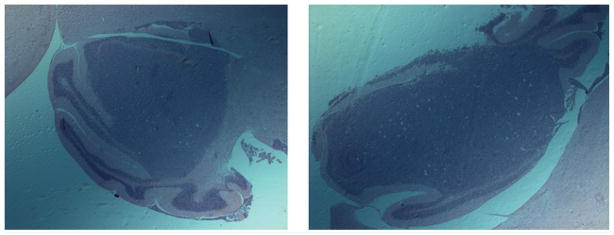
Hematoxylin and eosin staining confirms presence of MB-004 tumors in mouse cerebellum Tumor area (dark purple) shows clear morphological differences in cell density as compared to surrounding healthy cerebellar tissue (light purple).

Histology was performed in a subset of mice to confirm development of MB-004 tumors by hematoxylin and eosin (H&E) staining. High-density regions of small round tumor cells were easily visualized with clear boundaries, as well as presence of vasculature, which are indicative of tumor as compared to the rest of the stained brain (Figure 3).

**FIGURE 4:**
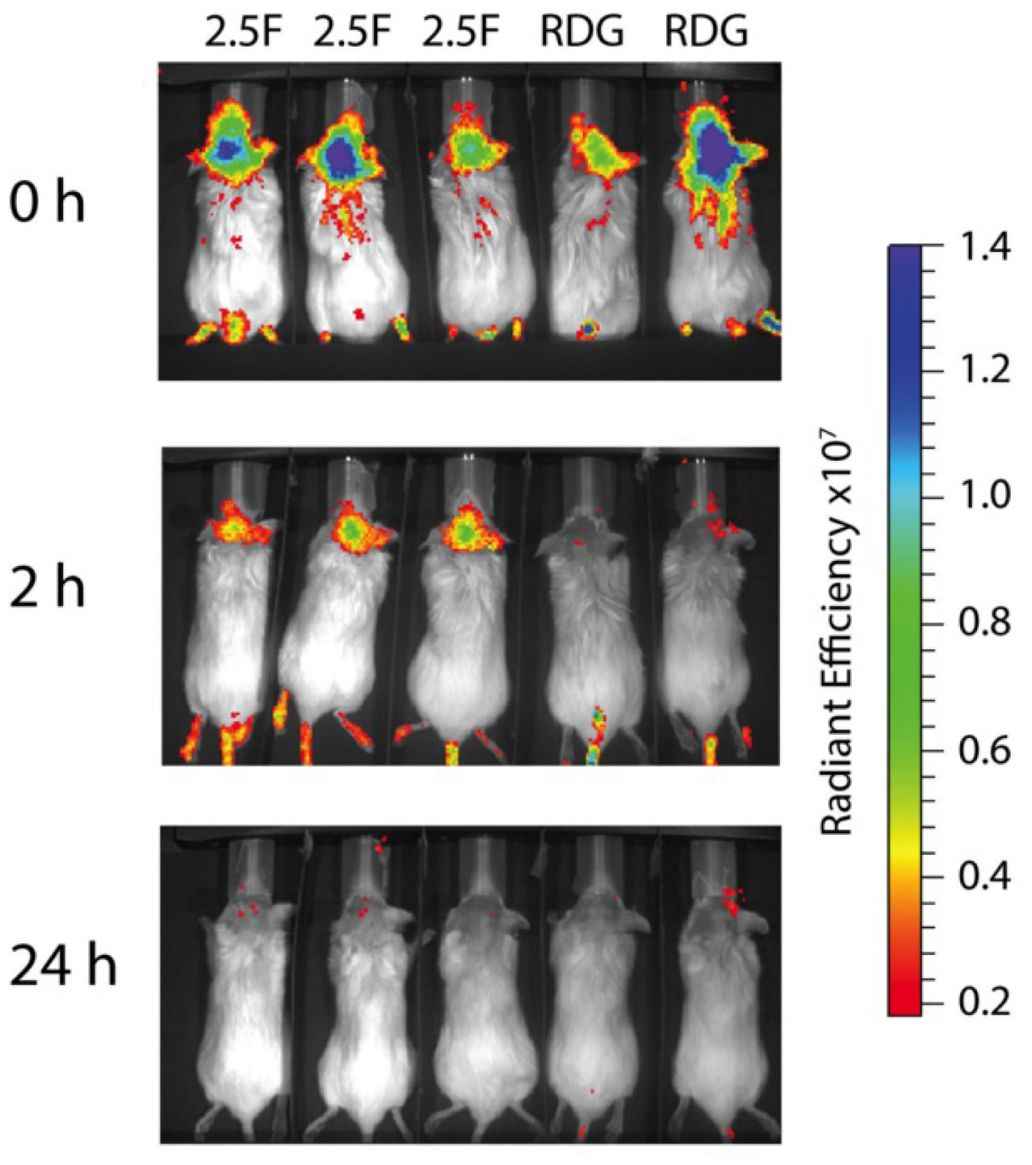
AF680-EETI 2.5F exhibits specific localization to MB-004 tumors Mice with orthotopically implanted intracranial MB-004 tumors were imaged with AF680-labeled knottins. Immediately post-administration, fluorescent signal was detected in all mice by near-infrared optical imaging. After two hrs, signal is localized to cerebellar tumors in mice injected with AF680-EETI 2.5F, but is cleared in mice injected with the non-binding AF680-EETI RDG control. Bound AF680-EETI 2.5F washes out of tumor tissue and is cleared after 24 hrs. Scale bar is reported in radiant efficiency: emission light (photons/sec/cm^2/str)/excitation light (μW/cm^2).

The ability of EETI 2.5F to target intracranial medulloblastoma was evaluated in mice bearing MB-004 tumors. Immediately following administration of AF680-EETI 2.5F or AF680-EETI RDG by tail vein injection, fluorescent signal was present in all mice indicating probe circulation through the body, as detected by non-invasive near-infrared fluorescence imaging using an IVIS Spectrum instrument (Figure 4). At two hrs post-administration, imaging signal from AF680-EETI 2.5F remained localized within the brain, and could be detected through the intact skull and skin (Figure 4). In contrast, lack of intracranial signal was observed from the negative control peptide AF680-EETI RDG, indicating that tumor targeting of AF680-EETI 2.5F is mediated by integrin-specific interactions, and that minimal background signal results from peptide vehicle and dye molecule alone. By 24 hrs, the AF680-EETI 2.5F signal cleared from the brain and was no longer detected by optical imaging (Figure 4).

**FIGURE 5:**
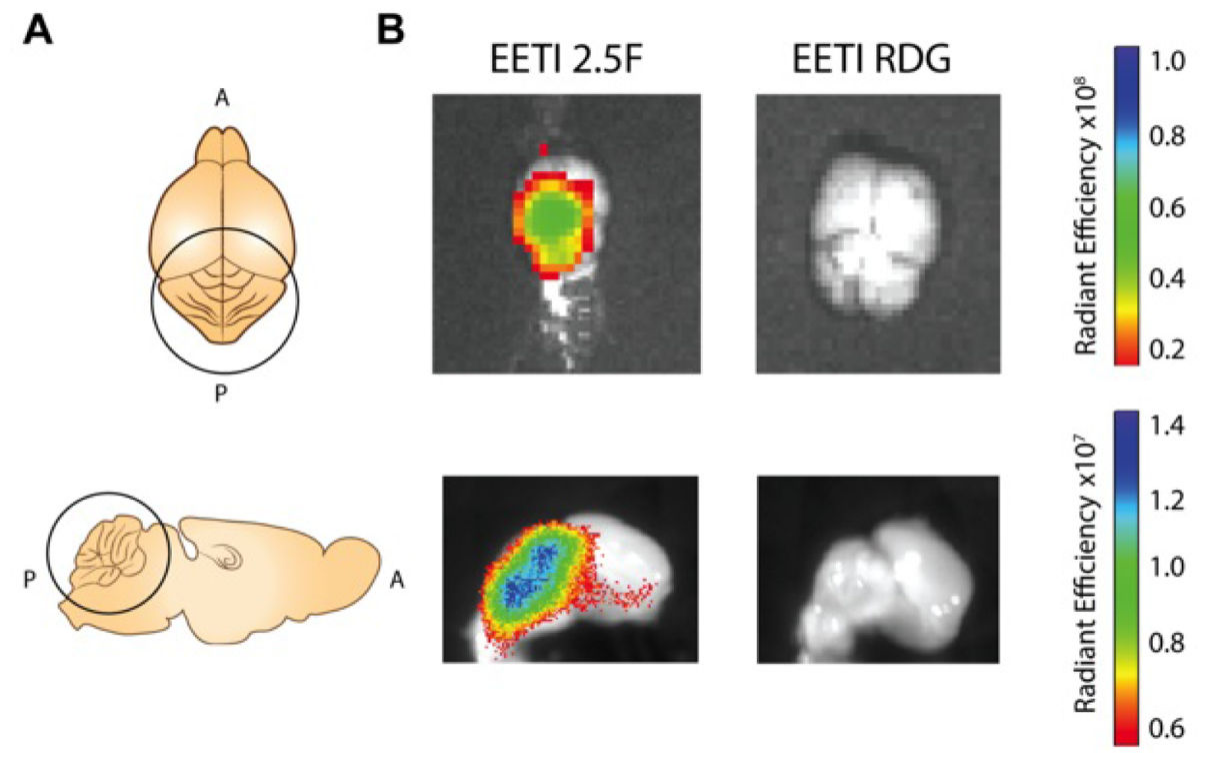
Ex-vivo imaging confirms cerebellar tumor localization (A) Schematic of murine brain anatomy. The top represents a superior view of brain and bottom represents the mid-sagittal view, with the cerebellum circled in both schematics (A=anterior, P=posterior). (B) Tumor-bearing mice were injected with AF680-EETI 2.5F or AF680-EETI RDG. At two hrs post-administration, whole-brains were excised and imaged (left panel). Fluorescent signal was observed in excised tissue from mice injected with AF680-EETI 2.5F. Brains were cut along the mid-sagittal plane and imaged (right panel) to show localization of signal in the cerebellum.

To confirm that the signal observed using non-invasive whole body imaging was localizing to the tumor, animals were sacrificed and brains were removed for *ex vivo* imaging analysis two hrs post-administration. The brains of animals that received AF680-EETI-2.5F showed imaging signal that localized specifically to the cerebellum region, while those receiving AF680-EETI-RDG showed no detectable imaging signal (Figure 5). Similarly, when brains were sliced along the mid-sagittal plane, all imaging signal was localized to the upper cerebellum region (Figure 5).

### Knottin EETI 2.5F can target intracranial tumors following IV and IP administration

**FIGURE 6:**
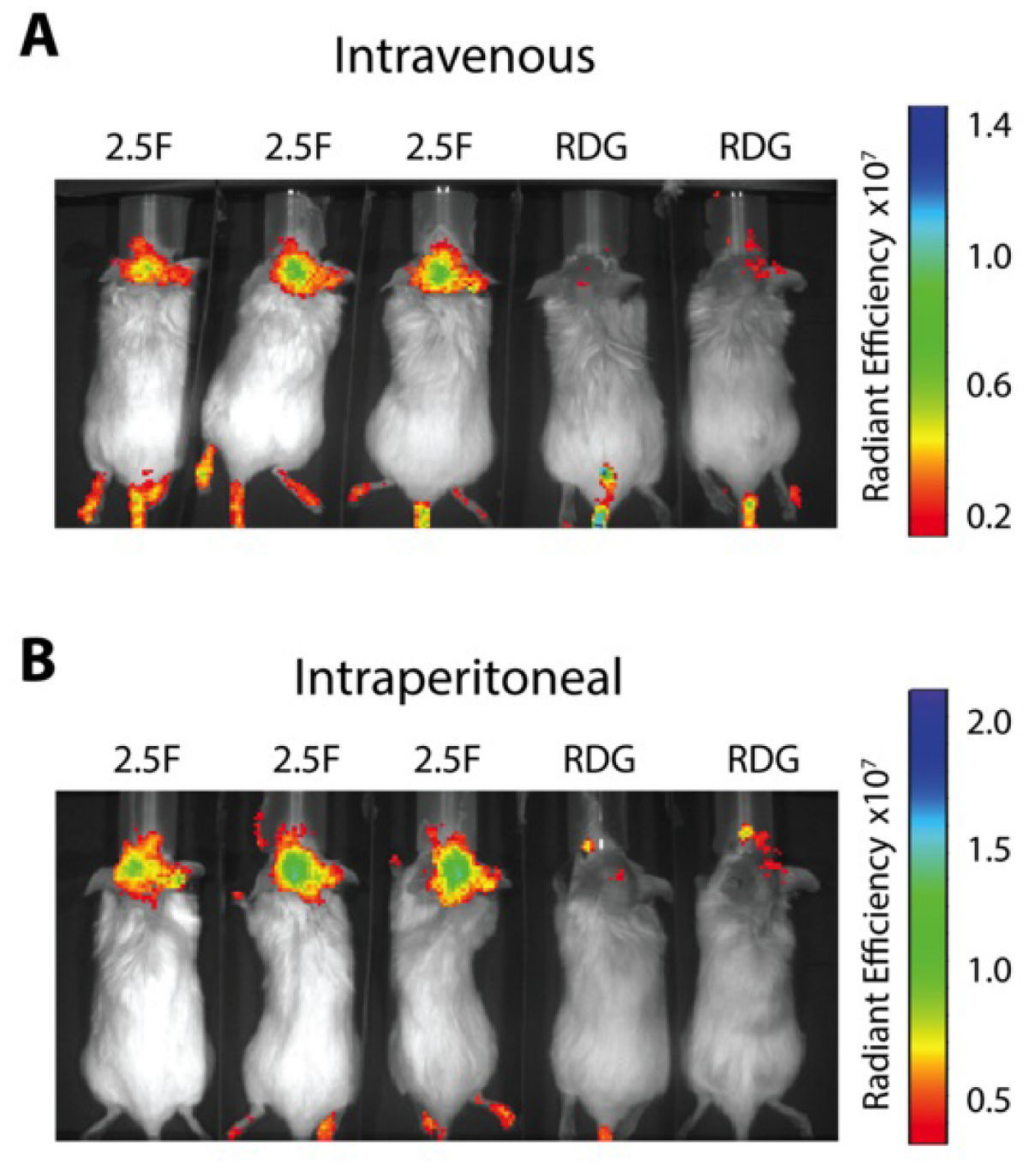
AF680-EETI 2.5F signal is observed upon intravenous or intraperitoneal administration Tumor-bearing mice were injected with AF680-EETI 2.5F or AF680-EETI RDG (A) via tail vein injection or (B) via intraperitoneal injection. Mice were imaged two hrs post-administration and tumor localization of AF680-EETI 2.5F was observed using both routes of administration.

Traditionally, most imaging agents are administered by intravenous injection, which in the case of murine models are delivered through the tail vein. We further evaluated if AF680-EETI 2.5F could specifically target patient-derived intracranial tumors if dosed through the intraperitoneal cavity. The same dose of labeled knottin peptides AF680-EETI 2.5F or AF680-EETI RDG were given via tail vein injection or intraperitoneal (IP) injection, and animals were imaged directly after and two hrs post-administration. Fluorescent signal was observed in mice of both groups immediately following injection. At two hrs post-administration, clearance of AF680-EETI RDG signal was observed, while we again saw specific accumulation of AF680-EETI 2.5F signal within the brain (Figure 6). This data indicates that brain tumor localization occurs following both intravenous and intraperitoneal administration routes.

## Discussion

Cystine-knot miniproteins, also known as knottins, have emerged as promising molecules for both diagnostic and therapeutic applications [31–32]. Their small size allows for increased tumor penetration as well as ideal pharmacokinetics (i.e. fast blood clearance) for use as diagnostic agents, compared to alternative tumor targeting proteins, such as antibodies [33–34]. The disulfide-bonded structure of knottins confers high thermal, chemical, and proteolytic stability [24–25], particularly to serum proteases [27], making them favorable for *in vivo* applications like tumor targeting. Knottins are thought to be non-immunogenic due to their fast clearance and high stability, which may allow them to avoid presentation to the immune system [35]. While our current study demonstrates tumor targeting through both intravenous and intraperitoneal administration routes, the high stability of knottins has also presented opportunities for oral delivery [25, 36–37].

In alternate approaches, researchers have exploited the inherent tumor-targeting ability of molecules found in nature to develop brain tumor imaging agents. Five-Aminolevulinic acid (5-ALA) is a natural compound that is approved by the FDA (as a topical solution) and the EMA in Europe (as an oral agent) for visualizing malignant gliomas during neurosurgical procedures through optical imaging [9, 38]. Five-ALA is the starting compound generated in the porphyrin synthesis pathway that leads to heme production in mammals and chlorophyll in plants [39]. Five-ALA has been shown to promote synthesis and accumulation of porphyrins, which are naturally fluorescent, in endothelial and cancerous tissue [40]. Another natural compound, chlorotoxin, isolated from the venom of the death-stalker scorpion, *Leiurus quinquestriatus,* has also been developed as a tumor targeting agent [41]. The molecular target for Tumor Paint is less clear and was thought to involve matrix metalloproteinase-2, although recent evidence suggests a role for Annexin A2 [42]. In a clinical study, a radiolabeled synthetic version of chlorotoxin was shown to target human glioma following intracavitary injection, with negligible toxicity [43]. When conjugated to a near-infrared dye, this peptide was given the moniker “Tumor Paint” for its ability to illuminate a host of tumors, such as glioma, medulloblastoma, and intestinal cancer, by spectral imaging in animal models [44–45]. A variant of Tumor Paint conjugated to indocyanine green (BLZ-100, Blaze Biosciences) is currently under clinical development for image-guided resection of tumors [46].

EETI 2.5F offers several potential benefits compared to alternative molecular imaging agents under development for image-guided tumor resection. Favorably low imaging signal has been consistently observed in non-target organs, such as kidney and liver [27, 33]; this feature of EETI 2.5F appears to be unique compared to other peptide and protein-based imaging probes [47]. Moreover, optimal tumor contrast (i.e. tumor-to-background levels of signal) is achieved with EETI 2.5F at two hrs post-administration in mice. In comparison, signals from Tumor Paint were imaged 24–48 hrs post-administration in mice [48], which, if necessary for optimal tumor contrast, may be less convenient for clinical use in humans. The clearance of EETI 2.5F from the tumor by 24 hrs post-administration can be a benefit or a limitation depending on the intended clinical application; however, in previous studies, we showed that robust brain tumor imaging signal in mice can still be observed six hrs post-administration [29], which may be an ideal time range for surgery.

For 5-ALA, in addition to challenges with timing of administration and dosing, limitations include rapid photobleaching and reliance upon non-specific heme metabolism for probe uptake in neoplastic tissue, which can vary across tumors [40]. For example, although 5-ALA has been shown effective in visualizing gliomas, its effectiveness in medulloblastomas has been mixed and may correlate with CD133 expression [49]. EETI 2.5F avoids the latter by targeting a panel of receptors that are broadly expressed on tumors. In contrast to 5-ALA, the spectral properties of the EETI 2.5F conjugate can be tuned by attachment of dyes that are compatible with surgical instruments and detection systems already used by neurosurgeons worldwide. For *in vivo* optical imaging applications, a probe with absorption and emission spectra in the near-infrared region is optimal, due to the inherent autofluorescence of tissue and blood. While our current study uses AF680, this dye has not yet been evaluated or approved for human use. Therefore, in future studies, we will conjugate EETI 2.5F to the FDA-approved near-infrared dye indocyanine green, and will evaluate this probe in pre-clinical tumor models to position this technology towards human translation.

In previous studies, we reported minimal AF680-EETI 2.5F brain tumor localization in mice receiving an injection of saline instead of tumor cells [29], indicating that the robust imaging signals observed are not a result of integrins expressed during injury or wound healing from orthotopic implantation surgery. The blood-brain barrier is a major obstacle for efficient delivery of molecules to the central nervous system due to tight cell-cell junctions that limit permeability as well as the presence of active efflux transporters [50–52]. Some molecules can access brain tumor tissue through a compromised blood-tumor barrier [51, 53–54] or through active transport mechanisms [53, 55]. The precise mechanism by which EETI 2.5F can access brain tumors is currently being investigated, but is an intriguing question to consider in light of data suggesting that Tumor Paint, which is also a knottin, can cross an intact murine blood-brain barrier [44].

A number of alternative peptides and peptidomimetics containing an RGD integrin-binding motif have been developed and used as molecular imaging agents [12, 56]. Many of these probes have shown limitations, such as weak integrin-binding affinity, low tumor imaging contrast, and inability to target brain tumors. One prominent example, a cyclic peptide containing an RGD motif [c(RGDyK)], was able to detect α_V_β_3_ integrin in subcutaneous glioblastoma tumors [57–59], but efforts to image intracranial tumors with this probe were not successful [29, 57]. EETI 2.5F is unique in that it is the only known peptide that binds with tight affinity to three prominent tumor-associated integrin receptors: α_V_β_3_, α_V_β_5_, and α_5_β_1_. High levels of at least one these three integrin subtypes have been found in many cancers, including glioma, melanoma, non-small cell lung cancer, and tumors of the prostate, ovary, breast, cervix, and pancreas [15], indicating that EETI 2.5F has potential to target a wide range of tumors for diagnostic and therapeutic applications. Towards this goal, in addition to medulloblastoma, EETI 2.5F has been used to image glioblastoma, lung, and ovarian tumors using a variety of modalities, including optical imaging [29], positron emission tomography [26–28], single-photon emission computed tomography [60], or ultrasound imaging [61]. Thus, EETI 2.5F not only has promise as a tool for delineating tumor boundaries in surgery, but also has potential utility as a companion diagnostic for disease staging and management.

## Conclusions

We demonstrate the ability of an engineered integrin-binding knottin peptide, EETI 2.5F, to specifically target intracranial medulloblastomas in a patient-derived xenograft model. Imaging signal resulting from IV or IP administration of EETI 2.5F, conjugated to a near-infrared dye, was robust and could be detected by non-invasive optical imaging. Although the data presented here is focused on medulloblastoma, EETI 2.5F will likely find utility in other tumor targeting applications as α_V_β_3_, α_V_β_5_, and α_5_β_1_ integrins targeted by EETI 2.5F are present on a wide variety of cancers. Collectively, these results motivate further development of EETI 2.5F as a tool for fluorescence-guided surgical resection of brain tumors.
